# From Bench to Bed: The Current Genome Editing Therapies for Glaucoma

**DOI:** 10.3389/fcell.2022.879957

**Published:** 2022-05-16

**Authors:** Meihui He, Rong Rong, Dan Ji, Xiaobo Xia

**Affiliations:** ^1^ Eye Center of Xiangya Hospital, Central South University, Changsha, China; ^2^ Hunan Key Laboratory of Ophthalmology, Changsha, China; ^3^ National Clinical Research Center for Geriatric Disorders, Xiangya Hospital, Central South University, Changsha, China

**Keywords:** glaucoma, gene therapy, CRISPR, eye, aqueous humor, optic nerve, retina

## Abstract

Glaucoma is a group of optic neuropathies featured by degeneration of retinal ganglion cells and loss of their axons in the optic nerve. The only currently approved therapies focus on lowering intraocular pressure with medication and surgery. Over the previous few decades, technological advances and research progress regarding pathogenesis has brought glaucomatous gene therapy to the forefront. In this review, we discuss the three current genome editing methods and potential disease mechanisms of glaucoma. We further summarize different genome editing strategies that are being developed to target a number of glaucoma-related genes and pathways from four aspects including strategies to lower intraocular pressure, neuroprotection, RGC and optic nerve neuro-regeneration, and other strategies. In summary, genome therapy is a promising therapy for treating patients with glaucoma and has great potential to be widely applied in clinical practice.

## Introduction

Glaucoma is a group of optic neuropathies featured by degeneration of retinal ganglion cells (RGCs) and loss of their axons in the optic nerve (Jonas et al., 2017). By 2020, there were an estimated 80 million glaucoma patients with approximately 11.2 million people being blind, making glaucoma a leading cause of irreversible blindness worldwide (Quigley and Broman, 2006; Cook and Foster, 2012). In addition, there are a tremendous number of asymptomatic early-stage individuals, which is supported by surveys revealing that only 10–50% of people with glaucoma are aware of the disease. As a result, by the time many individuals with glaucoma come to the hospital due to eye discomfort, they often have apparent optic nerve damage and irreversible loss of visual function.

Glaucoma is classified as primary glaucoma, secondary glaucoma and congenital glaucoma, based on pathogenesis, age of onset. Primary glaucoma is further classified as open-angle and angle-closure (or closed-angle) glaucoma, according to the trabecular meshwork (TM) function and iridocorneal angle. Increased cup‐disk ratio (CDR), CDR asymmetry, elevated intraocular pressure (IOP), specific genetic backgrounds, and reduced corneal thickness each raise the risk of primary glaucoma (Stein et al., 2021). Secondary glaucoma can result from trauma, tumors, use of corticosteroids, or inflammation. There are a number of secondary glaucoma variety including pigmentary, hemolytic, pseudoexfoliative, uveitic, neovascular, ciliary-block glaucoma and so on. Primary congenital glaucoma is caused by developmental abnormalities in the anterior segment and aqueous outflow pathway during fetal development. Among glaucoma, primary open-angle glaucoma (POAG) is the most common type worldwide.

Despite the great adverse impact of glaucoma on human health, there is still no adequate treatment for completely preventing glaucoma progression and no way currently to reverse the damage. Clinical treatments focus on lowering IOP using medication or surgery. However, risk factors beyond the elevation of IOP may aggravate glaucoma. Therefore, lowering IOP in many cases fails to halt further damage to RGCs and their axons. The extent and duration of medical and surgery therapy efficacy are limited.

With recent advancements in technology and the discovery of specific disease mechanisms, there is growing possibility that future glaucoma treatment will focus more on the direct protection of RGCs and the optic nerve and neuron regeneration to reverse glaucomatous injury. This potential propels gene therapy and genome editing into the spotlight of the glaucoma field as they can be tailored to target specific disease pathways and exert lasting and effective outcomes. Of the 22 gene therapy products that had been approved worldwide as of 2019 (Ma et al., 2020), Luxturna™ by Spark Therapeutics, Inc. was the first viral ocular gene therapy. Luxturna™received FDA approval in December 2017 to treat Leber congenital amaurosis type 2 (LCA2), which is an inherited retinal disease (IRD) caused by mutations in the RPE65 gene, leading to severely impaired vision at birth. In Luxturna™ therapy, RPE65 complementary DNA (cDNA) is administrated to the subretinal space of both eyes to treat the retinal dystrophy, which has achieved great vision improvement.

Gene therapy, historically defined as the transfer of genetic material to cells, has extended into three fields, gene augmentation, gene suppression, and genome editing, of which genome editing stands out for its characteristic of precise manipulation of targeted genes. Although antiviral and anti-cancer strategies account for most clinical trials of genome editing, there have been some clinical trials regarding ocular diseases (see in Table 1). The eyeball has the characteristics of self-sealing, the scope of influence after gene drug injection is small, and the eye to a certain extent is an immune privileged site. Clinical trials have shown that the use of adeno-associated virus (AAV) or lentiviral (LV) vectors to deliver gene therapy in the eye do not cause systemic side effects or a significant immune response against the vectors. Therefore, the application of gene therapy in the eye for genetic-based diseases has been the first to mature. Moreover, genome editing in ophthalmology is gaining momentum with the accumulation of promising advancements in preclinical studies (see in Table 2).

**TABLE 1:** Clinical trials investigating human genome editing for ocular diseases. Target means the diseases targeted in ophthalmology. Strategy means the experimental process by genome editing to treat the targeted disease.

Identifier	Phase	Title	Conditions	Intervention	Status
NCT04560790	Phase1/2	CRISPR/Cas9 mRNA Instantaneous Gene Editing Therapy Assisted Corneal Transplantation in the Treatment of Refractory Viral Keratitis	Viral KeratitisBlindness Eye	BD111 CRISPR/Cas9 mRNA Instantaneous Gene Editing Therapy	Recruiting
NCT01949324	Phase 2	A Phase 2 Multicenter Randomized Clinical Trial of Ciliary Neurotrophic Factor (CNTF) for Macular Telangiectasia Type 2 (MacTel)	Macular Telangiectasia Type 2	Ciliary neurotrophic factor released from NT-501 encapsulated cell implant	Completed
NCT02862938	Phase 2	Study of NT-501 Encapsulated Cell Therapy for Glaucoma Neuroprotection and Vision Restoration	glaucoma	Ciliary neurotrophic factor released from NT-501 encapsulated cell implant	Active
NCT04577300	Phase 2	Dual Intravitreal Implantation of NT-501 Encapsulated Cell Therapy for Glaucoma	glaucoma	Ciliary neurotrophic factor released from NT-501 encapsulated cell implant	Not yet recruiting
NCT03872479	Phase1/2	Open-Label, Single Ascending Dose Study to Evaluate the Safety, Tolerability, and Efficacy of EDIT-101 in Adult and Pediatric Participants with Leber Congenital Amaurosis Type 10 (LCA10)	Leber Congenital Amaurosis 10	EDIT-101 (subretinal injection), a candidate genome-editing therapeutic, to remove the aberrant splice donor created by the IVS26 mutation in the CEP290 gene and restore normal CEP290 expression	Recruiting

**TABLE 2:** Representative preclinical studies of gene editing for ocular diseases.

Target	Strategy	References
Leber congenital amaurosis type 10	Removal the aberrant splice donor created by the IVS26 mutation in the CEP290 gene	Maeder et al. (2019)
Meesmann’s epithelial corneal dystrophy	Allele-specificdisruption of KRT12-L1^32^P gene by CRISPR/Cas9	Courtney et al. (2016)
Fuchs’ endothelial corneal dystrophy	Reduction of intronic CTG triplet repeat expansion in the TCF4 gene by CRISPR/Cas9	Rong et al. (2020)
Retinitis pigmentosa	Correctionof the Pde6b-rd1 mutation in the mouse retina	Wu et al. (2016)
Retinitis pigmentosa	Disruption of dominant mutation inRho-S334 gene	Bakondi et al. (2016)
Retinitis pigmentosa	Inserting a copy of Mertk exon 2 into intron 1	Suzuki et al. (2016)
Laser-induced choroid neovascularization	Edition of genomic Vegfaand Hif1a in vivowhich abolished angiogenesis	Kim et al. (2017)
Autosomal dominant cone-rod dystrophy (CORD6)	Disruption of GUCY2D to alter retinal function and structure by CRISPR/Cas9	McCullough et al. (2019)
Leber congenital amaurosis	Correction of a disease-associated nonsense mutation in Rpe65 in rd12 mice by CRISPR-Cas9	Jo et al. (2019)
X-linked juvenile retinoschisis	Correction of the disease-associated RS1-C625T mutation in a 3D retinal organoid by CRISPR/Cas9	Huang et al. (2019), Yang et al. (2020)
X-linked juvenile retinoschisis	Knocking in of the RS1 gene with the homology-independent targeted integration (HITI) strategy by CRISPR/Cas9	Chou et al. (2020)
Usher syndrome type II	Deletion of the exon 12 of mouse Ush2a gene (corresponding to exon 13 of human USH2A) using CRISPR/Cas9-based exon-skipping approach	Pendse et al. (2019)
Usher syndrome (USH) type III	Excision of the mutated intronic CLRN1 splicing mutation	Panagiotopoulos et al. (2020)
Non-disease condition	Knockout of both PXDN by CRISPR in mice showed completely or almost closed eyelids with small eyes, having no apparent external morphological defects in other organs	Kim et al. (2019)
Enhanced S-cone syndrome	Correction of disease-causing NR2E3 mutations in patient-derived induced pluripotent stem cells (iPSCs) by CRISPR/Cas9	Bohrer et al. (2019)
Non-disease condition	11-base pair deletions to the homologous PMEL in zebrafish by CRISPR/Cas9 caused profound pigmentation defects (Pigmentary glaucoma in human)	Lahola-Chomiak et al. (2019)
Glaucoma	Disruption of mutant MYOC by CRISPR/Cas9 in cultured human trabecular meshwork cells resulted in lower IOP and prevents further glaucomatous damage	Jain et al. (2017)
Glaucoma	Disruption Aquaporin 1 resulted in reduced IOP in treated eyes by CRISPR/Cas9	Wu et al. (2020)
Glaucoma	CRISPR-Cas9-mediated connective tissue growth factor (CTGF) suppression reduced glaucoma filtration surgery (GFS) fibrosisand improved human GFS outcomes	Lee et al. (2020)
Non-disease condition	RGCs differentiated from OPTN(E50K) mutated hPSCs by CRISPR/Cas9 exhibitednumerous neurodegenerative defects (glaucoma)	VanderWall et al. (2020)
Inherited retinal diseases	Correction of nonsense mutation in the Rpe65 gene regained retinal and visual functions	Suh et al. (2020)
Aniridia	Germline correction of the Pax6 small eye(Sey) mutation alone rescues the mutant phenotype	Mirjalili Mohanna et al. (2020)
Best disease, a dominant macular dystrophy	Normalization of BEST1 channel activity by CRISPR-Cas9 editing of the mutant allele	Sinha et al. (2020)

## Genome Editing Methods

Since the discovery of genes being the basic genetic unit that controls biological traits, it has become an aspiration of humans to modify them in order to cure diseases fundamentally. Gene therapy is the optimal indicated approach for diseases rooted in mutated genes. The areas in which gene therapy has been most commonly applied include cancers, monogenic diseases, cardiovascular diseases, infectious diseases, neurological diseases, and ocular diseases, among others. Gene therapy to date can be characterized as the knock-down of deleterious genes and augmentation of necessary or desirable genes and has included the genetic modification of mutated genes using site-specific editing.

### Endogenous DNA Repair Mechanisms

The realization that a targeted DNA double-strand break (DSB) can stimulate endogenous DNA repair mechanisms forms the foundation of genome editing. There are two main types of DNA repair, homology-directed repair (HDR) and non-homologous end-joining (NHEJ). HDR utilizes templates from either exogenously supplied donor sequence or sister chromatid for precise DNA repair, which leads to the insertion and correction of the relevant gene/DNA. In contrast to the predictable gene-editing that results from HDR, NHEJ functions to repair DSBs in a template-independent manner through direct ligation of DNA ends. This process is error-prone and has a high possibility of introducing insertions and/or deletions (indels) at the site of the break. These indels may cause gene mutations leading to frameshifts and premature stop codons. It is possible to cause gene deletions through NHEJ with large DNA segments.

To stimulate endogenous cellular DNA repair, current genome editing methods focus on various types of sequence-specific nucleases that form site-specific DSBs. For genome editing, novel nucleases are used, including zinc finger nucleases (ZFNs), transcription activator-like effector nucleases (TALENs), and clustered regularly interspaced short palindromic repeats (CRISPR)/Cas9.

#### ZFNs

The technology using ZFNs for genome editing was made possible with the discovery of the precise DNA-binding domain and FokI restriction endonuclease. The zinc finger protein is a type of transcription factor with the zinc finger domain forming the basis for the necessary DNA-binding specificity. The modular design of DNA-binding proteins makes it relatively easy to generate chimeric sequence-specific nucleases by replacing the FokI DNA-binding domain with a zinc finger domain.

#### TALENs

Similar to ZFNs, TALENs are programmable DNA-binding nucleases with the catalytic domain of the FokI endonuclease fused to transcription activator-like effector (TALE) repeats. Different from that of ZFNs, the highly conserved 34 amino acid TALE repeats are responsible for the DNA-binding specificities of TALENs instead of zinc finger domains. Each TALE repeat specifies a single base pair and makes it possible to target any DNA sequence of choice.

#### CRISPR/Ca9

CRISPR/Ca9 technology was initially derived from an adaptive immune system in bacteria that is used to defend against invading viruses. Among the main components of the technology, CRISPR RNAs (crRNAs) and trans-activating crRNAs (tracrRNAs) recognize specific DNA base pairs and the CRISPR-associated (Cas) proteins act as nucleases to perform precise cleavage of the DNA. After *in vitro* modification, the CRISPR/Ca9 has been simplified to two components by fusing the crRNAs and tracrRNAs as guide RNAs (gRNAs). The CRISPR/Cas system also requires a protospacer-adjacent motif (PAM) situated immediately 3′ to the target site. Cas9 has six domains of which the PAM interacting domain specifically recognizes PAM to initiate the targeted binding to the DNA. The Rec I domain of Cas9 acts as the gRNA binding domain. Domains HNH and RuvC are nuclease domains that cut single-stranded DNA. Once Cas9 finds a specific PAM, the gRNA attempts to pair with the target DNA sequence and consequently forms a DSB. Compared with the nuclease systems discussed above, Cas9 complexed with gRNAs is free of novel chimeric nucleases. Instead, the target sites are altered by simply modifying a few base pairs of the gRNAs.

Scientists today have recognized the potential ability to control genetic mutations using powerful biotechnology to modify the DNA in living cells and even modify the genetic code of all species on the planet. In addition to the NHEJ and HDR strategies, many novel CRISPR strategies have been developed. Among the many gene-editing tools, the latest and possibly most effective is CRISPR-Cas9. The development, transformation, and application of gene editing tools based on the CRISPR system have shown explosive growth, which allows for precise gene editing to better serve humans. The discovery of new Cas orthologues and variants, such as VRER SpCas9, VQR SpCas9 (Anders et al., 2016; Hu et al., 2018), Cas13 (Abudayyeh et al., 2017) and xCas9 (Hu et al., 2018), even further broadens the scope of recognition sequences in the genome and increases editing specificity.

**FIGURE 1:**
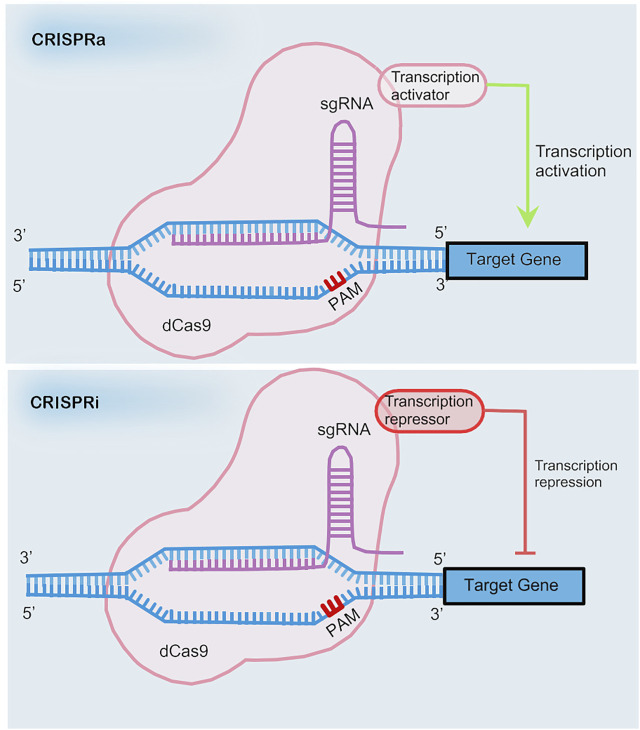
The mechanism of CRISPRa and CRISPRi.

CRISPR interference (CRISPRi) and CRISPR activation (CRISPRa) are types of safe pattern reformative CRISPR systems that avoid permanent sequence mutations (Dominguez et al., 2016) (see in Figure 1). Gene regulation can be achieved through a transcription depressor or transcription activator being fused with a nuclease-deficient Cas9 (dCas9). The fusion of dCas9 and the Krüppel associated box (KRAB) contributes to the down-regulation of transcription by binding with the promoter or downstream of the transcription start site via the guidance of single guide RNA (sgRNA). In the same way, a complex comprised of dCas9 and the p65 transactivating subunit of NF-kappa B or the transcriptional activation domain VP64 promotes the up-regulation of transcription.

**FIGURE 2:**
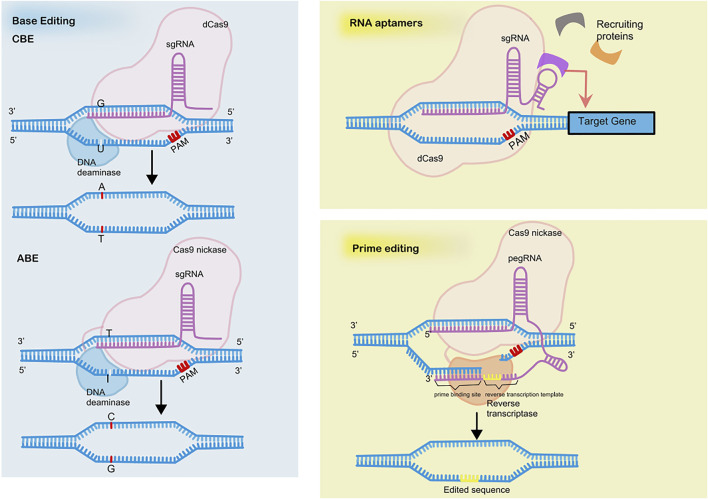
The mechanisms of base editing, prime editing and RNA aptamers.

However, application of CRISPRi is limited due to the large sizes of the coding sequences of dCas9 fusion proteins. Incorporation of RNA-protein interacting systems into gRNA helps resolve the limitation by recruiting effector proteins. The recruitment of RNA-binding proteins by RNA aptamers allows for the independent regulation of multiple genes simultaneously (Zalatan et al., 2015) (see in Figure 2).

Homology-independent targeting integration is a novel evolution of the NHEJ pathway, which directly ligates exogenous DNA fragments to DSBs. DSBs are generated in the genome targeting sequence, as well as both ends of the inserted DNA fragments, due to a pair of flanking CRISPR targeting sequences of the inserted DNA matching the genome targeting sequence (Suzuki et al., 2016).

The novel genome editing approach of base editing achieves precise base mutations without creating DSBs or the need of delivering templates (see in Figure 2). The base editor is a fusion of dCas9 with DNA deaminase plus DNA repairing proteins, if necessary. Two types of base editors have been developed, cytosine base editors (CBEs) and adenine base editors (ABEs), which allow the conversion of cytosine (C) to thymine (T) and adenine (A) to guanine (G), respectively (Komor et al., 2016; Gaudelli et al., 2017). The nucleotide position that can be effectively edited is called the active window and is located at positions four to eight downstream of the pre-interval sequence. Cas9 nickase is an H840A mutant of Cas9 and cleaves only the PAM-containing DNA strand. The innovation of combining uracil-DNA glycosylase inhibitor (UGI) and Cas9 nickase to cleave the unedited complementary strand vastly increases editing efficiency.

A brand-new genome editing tool based on CRSIPR is prime editing, which is reported to repair 89% of all 75,000 pathogenic human genetic variations (Anzalone et al., 2019) (see in Figure 2). Prime editing enables 12 kinds of base-to-base conversions, insertions up to 44 bases long, and deletions up to 80 bases long. The prime editor (PE) consists of a reverse transcriptase fused to a Cas9nickase and a gRNA with added RNA sequence at 3′ ends called prime editing guide RNA (pegRNA). The RNA sequence at 3′ ends includes a primer binding site to initiate reverse transcription and serves as a template for reverse transcription.

**FIGURE 3:**
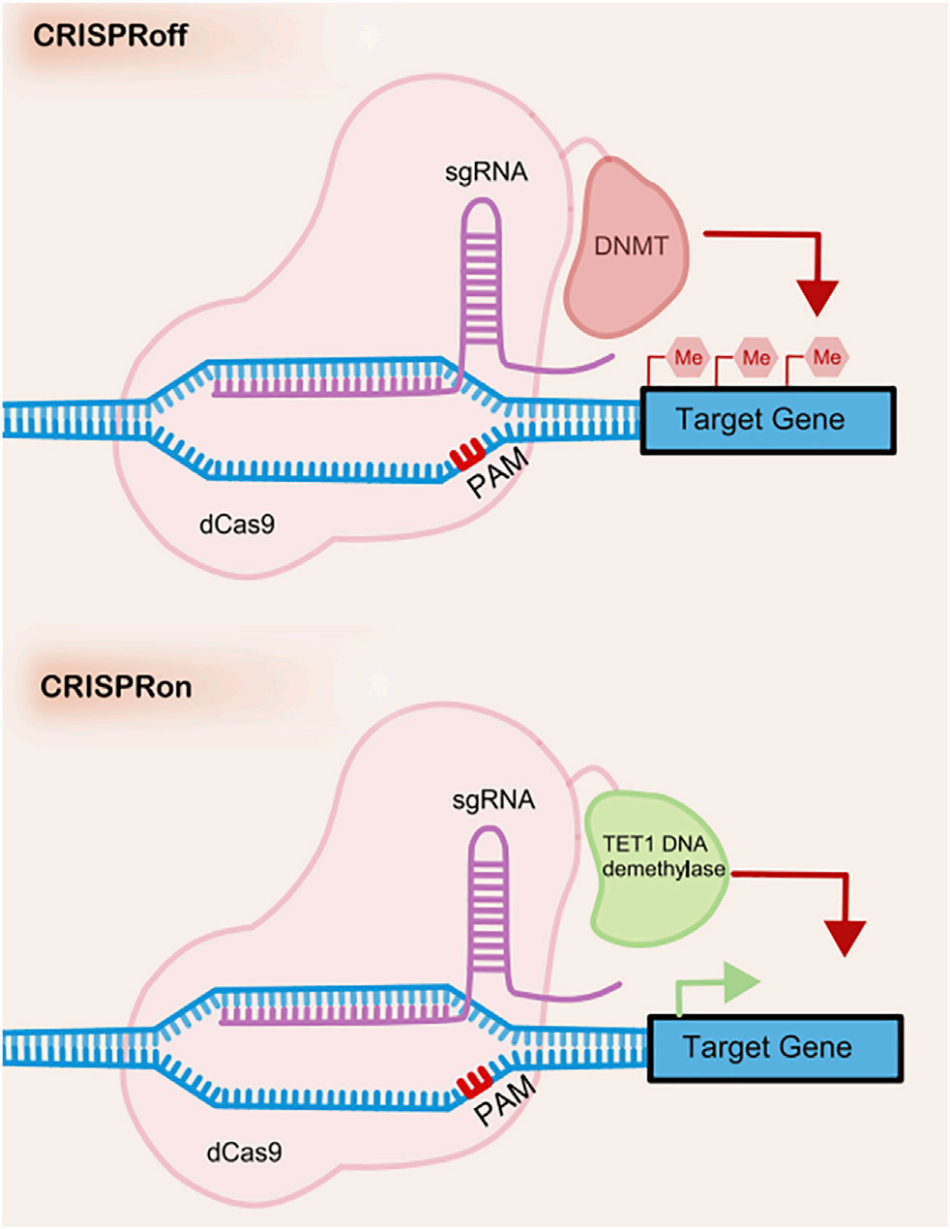
The mechanism of CRISPRoff and CRISPRon.

CRISPR off is a reversible and inheritable epigenetic memory editor based on the CRISPR system (Nuñez et al., 2021) (see in Figure 3). With guidance from gRNA, CRISPR off promotes the methylation of the targeting DNA to repress gene transcription without the generation of DSBs. Furthermore, the epigenetic memory by CRISPR off can be reversed by CRISPR on, which applies demethylase to relieve the transcription repression. This method can even silence genes that do not have large methylated regions (CpG islands), which significantly broadens the scope of its application.

### Gene Delivery Vectors

Currently, adenoviruses, adeno-associated viruses (AAVs), and lentiviral vectors represent the majority of viral vectors used for gene therapy. AAV is a group of replication-defective, non-pathogenic virus containing a single-stranded DNA genome comprised of 4.7 kilo nucleotides (4.7knt) (Srivastava et al., 1983). Due to their non-pathogenicity, low immunogenicity and the ability of mediating persistent transgene expression, AAV vectors are currently the most widely used and the most efficient vehicle for *in vivo* gene delivery (Gaj et al., 2016). The availability of 13 AAV serotypes and hundreds of variants has greatly expanded the scope and speed of transduction (Wang et al., 2019). However, the main drawback of AAV is the limited packaging capacity which is less than ∼4.8 kb of DNA. This has posed a challenge for the delivery of large nucleases such as TALENs and CRISPR-Cas9, which has to be delivered using a dual-AAV approach.

Compared to AAVs, adenoviral vectors have larger genome size (∼30–40 kb pairs) so as to deliver much larger transgenes (Maggio et al., 2014). Adenoviral vectors do not integrate to the genome but can achieve persistent expression using its variants (Wang et al., 2019). In a glaucoma gene therapy study, adenovirus five vectors were used for delivery of CRISPR/SpCas9 system to knock out human MYOC gene (Jain et al., 2017). However, one limitation of adenoviruses is their relatively high immunogenicity and the high prevalence of neutralizing antibodies in population, resulting in the limited application for ocular gene therapy (Yang et al., 1996).

Lentivirus is also a promising system for delivery of transgenes. Lentiviral vector gene–carrying capacity (8–10 kb) is between that of adenoviruses and AAVs, allowing for the delivery of most transgenes (Balaggan and Ali, 2012). Lentiviruses can achieve persistent gene expression by integrating to the genome, which may also cause risk of insertional mutagenesis (Romano, 2012). Lentiviruses have been applied in several ocular gene therapies, where they are able to transduce trabecular meshwork (Khare et al., 2008), RPE cells (Yin et al., 2022), photoreceptor cells (Hanke-Gogokhia et al., 2021), Müller cells and ganglion cells (Zhou et al., 2021). The lower transducing efficiency in ocular gene delivery limited the clinical application.

Recently, the cell-specific targeting of these gene vectors has greatly advanced to pave the way for successful clinical trials in future. The current strategies of cell-specific targeting include modifying tropism of delivery vectors, designing to target the specific molecular markers and carrying cell-specific promoter in the viruses (Hulliger et al., 2020; Johari et al., 2021). A previous study found that due to the high expression of heparin sulfate proteoglycan in RGCs, which mediates attachment to AAV2, AAV2 has a high tropism for RGCs (Summerford and Samulski, 1998). Another example is an optimized hypoxia regulated, RPE cell-specific gene therapy to inhibit choroidal neovascularization (Biswal et al., 2018). Researchers achieved production of human endostatin (a powerful angiostatic protein) in RPE through AAV2, which comprised a RPE-specific promoter and HIF-1 response elements (HRE).

## Pathogenesis of Glaucoma

### Genetics

It is well known that glaucoma is markedly affected by genetic factors and is a complex genetic disease (Aboobakar and Wiggs, 2022). There has been evidence suggesting that small variations, including single nucleotide polymorphisms (SNPs) may be underlying cause of glaucoma. Besides, these SNPs may play highly pathogenic, mildly pathogenic or protective role in causing glaucoma. Genome-wide association studies (GWAS) is a genome-wide method that compares the genetic profile of SNPs between glaucoma cases and normal groups, aimed at identifying glaucoma-associated genomic regions (Aboobakar and Wiggs, 2022). Thus far, findings of GWAS have implicated 127 genetic loci that show strong associations with primary open-angle glaucoma (Gharahkhani et al., 2021). Among them, only four pathogenic genes, MYOC, NTF4, OPTN and WDR36 have been definitively linked to POAG. Similarly, multiple GWASs have been performed for PACG and 13 loci strongly associated with risk for developing PACG have been identified (Khor et al., 2016). Except for POAG and PACG, primary congenital glaucoma (PCG), which has significant genetic basis, has been identified five distinct loci through linkage analyses (Souma et al., 2016). Generally speaking, glaucoma is a complex polygenetic disease. Multiple genes with small effect sizes and possible environmental influences are necessary for disease pathogenesis.

### Glaucoma-Related Changes in the TM

Elevated IOP, which is commonly identified in glaucoma, is caused by an imbalance between the production of aqueous humor by ciliary epithelial cells and its drainage mainly through the TM or to a lesser extent through the uveoscleral outflow pathway. In patients with POAG, increased resistance to aqueous humor outflow through the TM is responsible for the elevation of IOP. The TM is a series of fenestrated beams and sheets of extracellular matrix (ECM) covered with endothelial-like trabeculocytes. There is a growing consensus that the TM plays a central role in the pathogenesis of glaucoma. Many changes have been elucidated in the TM structure and function regarding glaucoma.

Disturbances in extracellular matrix (ECM) homeostasis are known to occur in glaucomatous TM, but the mechanism remains unclear. Studies of transforming growth factor-beta 2 (TGFβ2) have revealed its effects on increasing cross-linking enzymes and ECM deposition and a potential glaucoma-TGFβ2 relationship (Wallace et al., 2013; Pattabiraman et al., 2014). Other explanations for the accumulation of ECM also exist, including the abnormal endocytic recycling of ECM components. As they are associated with glaucoma, caveolin-1 (CAV-1) and caveolin−2(CAV-2) are significant endocytosis-related proteins and their knockdown or mutation contributes to increased levels of ECM components and altered aqueous humor outflow rates (Loomis et al., 2014).

In addition to ECM abnormalities, studies and gene analysis of human TM cells have identified changes in cellular metabolism and expression levels of some genes. For instance, oxidative stress detected in TM epithelium cells is involved in early stage of glaucoma. The free radicals cause damage to the TM epithelium cells, which consequently leads to impaired outflow capabilities (Joe et al., 2003). Furthermore, the attack by free radicals on oxide-sensitive mitochondrial DNA causes mitochondrial dysfunction. The MYOC gene encodes myocilin and a MYOC mutation is known to impair mitochondria function in glaucomatous TM cells (He et al., 2009). Furthermore, MYOC is one of the pathogenic genes definitely linked to glaucoma. It is reported that the MYOC mutation is detected in 2–4% of POAG cases. However, the role of the MYOC mutation in glaucoma remains elusive. One hypothesis is that mutant myocilin is involved in mitochondrial depolarization and subsequent calcium overload, which leads to endothelial dysfunction in the TM. Another hypothesis includes intracellular aggregation of misfolded myocilin in the endoplasmic reticulum, which then leads to endoplasmic reticulum stress and potential cytotoxicity in TM cells (Joe et al., 2003).

### Glaucoma-Related Changes in RGCs

Common to all kinds of glaucoma is the degeneration of RGCs and optic neuropathy. Increased IOP is the main risk factor for glaucoma progression and the acceleration of optic neuropathy. Increased IOP leads to the eventual compression, deformation, and remodeling of the lamina cribrosa with mechanical axonal damage and disrupted bidirectional axonal transport within the optic nerve leading to neurotrophin deprivation. For instance, the retrograde transport of the neurotrophin brain-derived neurotrophic factor (BDNF) is blocked in RGCs (Quigley et al., 2000). Even with effective IOP control, the RGCs continue to degenerate. The mechanism by which RGCs die remains unknown. In addition to increased IOP, other mechanisms may include low ocular perfusion pressure (Zheng et al., 2010), apoptosis (Cordeiro et al., 2004), altered immunity, inflammation, excitotoxicity, and oxidative stress (Tezel, 2006), as well as excessive intracellular calcium and changes in glial cells (Rojas et al., 2014). Several genes are associated with glaucomatous RGC damage, such as optineurin (OPTN). OPTN is widely expressed in RGCs, has been identified as an autophagy receptor, and interacts with many proteins. Mutation of OPTN, such as E50K-OPTN, results in functional defects of vesicle trafficking and autophagy, leading to the death of RGCs by apoptosis (Sirohi and Swarup, 2016). Other mutations are also likely to cause glaucoma via different pathogenic mechanisms (Bansal et al., 2015).

RGCs do not have the capacity for self-renewal and self-repair following their degeneration and death. It has been suggested that a combination of intrinsic cellular properties and environmental factors limits the repair and regeneration of the optic nerve. Phosphatase and tensin homolog (PTEN) is a negative regulator of the mammalian target of rapamycin (mTOR) pathway and may account for the intrinsic inability of central nervous system axons to regenerate (Park et al., 2008). As for environmental factors, excessive myelin within the optic nerve (Wang et al., 2002) and reactive glial scarring and inflammation serve as a mechanical barrier to axonal growth. A study found that inhibition of microRNA miR-21 ameliorates excessive astrocyte activation and glial scar formation, which consequently promotes axonal regeneration (Li et al., 2018). Based on the information available, there has been an increasing consensus regarding the importance of neuroprotection in treating glaucoma fundamentally.

### Impact of the Biomechanical Property of Fibrous Layer

Considerable evidence indicates that deformation, remodeling, and mechanical failure of the fibrous layer, consisting of the cornea, sclera, and lamina cribrosa, is associated with glaucoma susceptibility and progression (Yang et al., 2017). Animal models provide the ability to evaluate the effects of alterations in the ocular connective tissues on glaucoma progression. Alteration in the biomechanical property of the cornea is an important risk factor for glaucoma progression in humans (Rahman et al., 2020). Scleral weakness with low fibrous density attributable to mutations in a disintegrin and metalloproteinase domain with thrombospondin type-1 motifs (ADAMTS10) can slow the course of glaucoma progression following increased IOP (Palko et al., 2013). It has also been reported that treatment of the sclera with cross-linking agents makes glaucomatous RGC axon damage worse (Kimball et al., 2014). Another study has revealed that peripapillary scleral stiffening reduces the biomechanical tension within the lamina cribrosa and exerts a neuroprotective effect (Coudrillier et al., 2016). Experimental studies have demonstrated that a chronic increase in IOP results in stiffness of the peripapillary sclera and remodeling of the collagen structure of the sclera. However, whether glaucoma-related scleral changes are protective or damaging is currently unknown. In general, the sclera is a dynamic structure and altering its structure and behavior in response to IOP changes may provide new treatment targets; however, this requires further research.

## Genome Editing of Glaucoma

**FIGURE 4:**
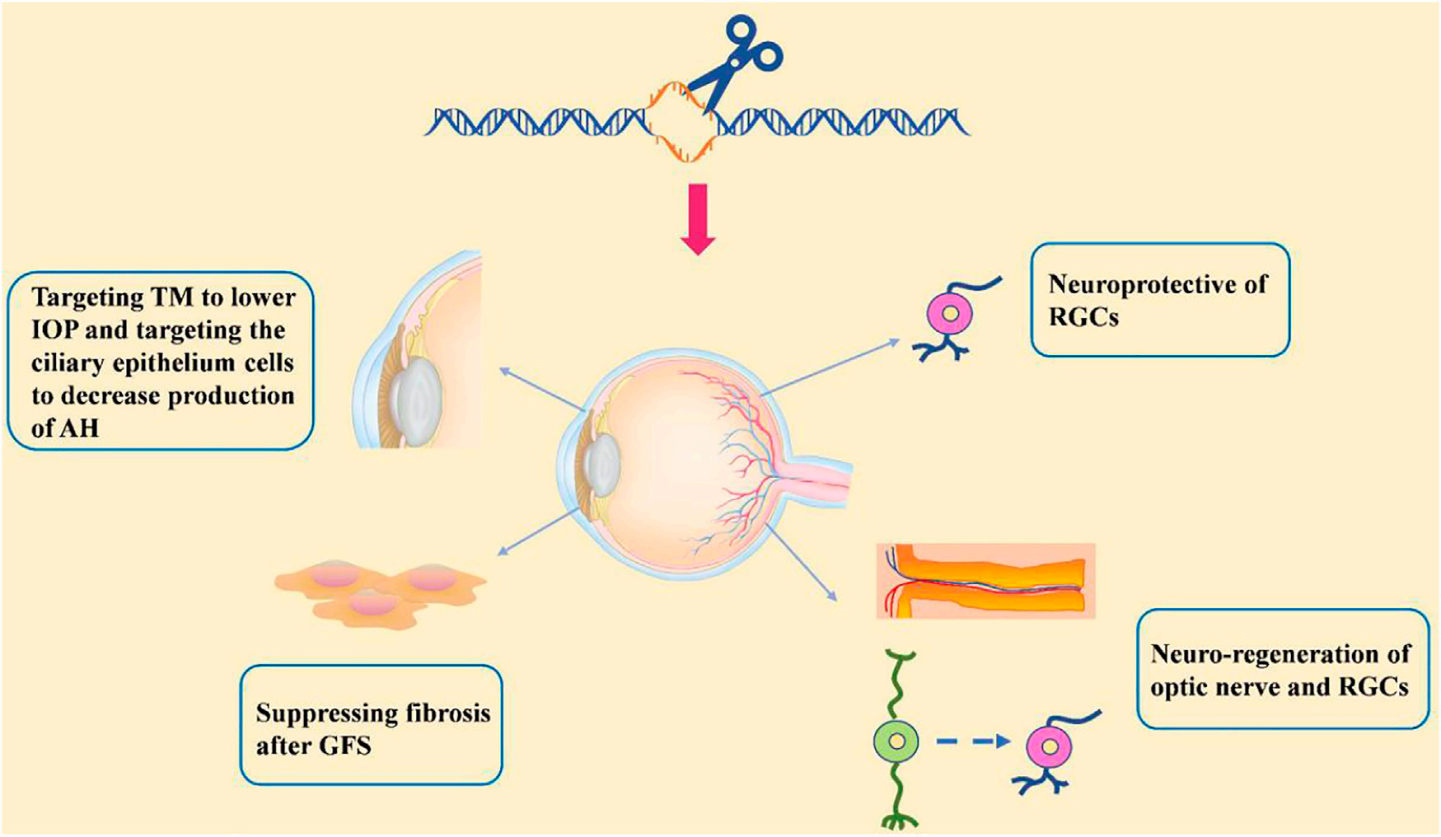
An overview illustration of the review.

Progress based on studies of the pathogenic mechanisms of glaucoma makes the possibility of gene therapy more viable. Gene therapy in treating glaucoma would be a great improvement over that of daily eye drops and surgery and would allow for more effective, targeted, and fundamental therapeutic outcomes following a one-time injection of the vector. Among gene therapy approaches, genome editing stands out for its characteristic of allowing the precise manipulation of target genes. Here, we discuss the recent advancements in genome editing for treating glaucoma (see in Figure 4).

### TM Targeting to Lower IOP

Impaired TM may cause resistance for aqueous humor drainage and subsequently lead to elevated IOP. Accordingly, therapy that targets the TM is attracting attention. In a recent study, the CRISPR/Cas9 system was used to disrupt the mutant MYOC gene in human and mouse TM cells and Ad5-crMYOC was intravitreally injected into transgenic POAG mice expressing mutant myocilin (Tg-MYOC^Y437H^) (Jain et al., 2017). Ad5-crMYOC treatment is able to increase the aqueous humor outflow rate, prevent IOP elevation, and improve RGC function. This indicates that disrupting mutant MYOC leads to improved TM cell function and prevents further glaucomatous damage (Jain et al., 2017). In addition to repairing damaged TM through gene therapy, advancements in stem cell therapy provides the ability to transfer stem cell-derived TM cells to the anterior chamber for glaucoma therapy. Transplantation of induced pluripotent stem cell (iPSC)-derived TM cells restores TM cellularity and function and promotes the proliferation of endogenous TM cells in both young and aged POAG mice in the Tg-MYOC^Y437H^ transgenic mouse model (Zhu et al., 2017; Zhu et al., 2020).

Expect for TM, the aqueous fluid outflow through the Schlemm’s canal and distal vessels. The angiopoietin (ANGPT)-TEK (tunica interna endothelial cell kinase) system is an endothelial growth factor pathway and both of ANGPT and TEK are highly expressed by SC endothelial cells. Studies have found that delivery of a recombinant ANGPT1-mimetic promoted developmental SC expansion in healthy and Angpt1 deficient eyes, suppressed intraocular pressure (IOP) elevation and RGC loss in a mouse model of PCG (Thomson et al., 2021).

### Other Strategies to Lower IOP

Based on the mechanism of IOP elevation, other strategies have been used to lower IOP, including the targeting of ciliary epithelium cells to decrease aqueous humor production and the use of trabecular bypass to increase the aqueous humor outflow capacity. In general, most glaucoma treatments focus on two main ways to lower IOP in effort to control the progression of glaucoma, increasing aqueous humor drainage and decreasing aqueous humor production by the ciliary body epithelium. Aquaporins are a family of water-transporting trans-membrane proteins and play a significant role in the formation of aqueous humor. Efficient aquaporin 1 (Aqp1) disruption by CRISPR/Cas9 in the mouse ciliary body epithelium following intravitreal injection is able to lower IOP and prevent RGC loss in a micro-bead glaucoma mouse model (Wu et al., 2020).

Glaucoma filtration surgery (GFS) is a common choice for controlling IOP. Aqueous humor drainage is increased through GFS, typically by diversion of the drainage under the conjunctiva and the formation of a filtration bleb. However, surgery failures commonly occur due to excessive sub-conjunctival fibrosis at the filtration bleb. Connective tissue growth factor (CTGF) is responsible for the fibrogenic reaction induced by fibroblasts; therefore, targeting CTGF to suppress fibrosis would potentially be an effective treatment to facilitate GFS success (Yamanaka et al., 2008). Permanent knockout of the CTGF gene using CRISPR/Cas9 system was performed by injecting viral vectors into the sub-conjunctival tissues of animals in a GFS rabbit model (Lee et al., 2020). It was demonstrated that disruption of CTGF promotes survival of the filtering blebs, improves bleb function, and reduces the overall degree of sub-conjunctival fibrosis.

### Neuroprotection

Novel neuroprotective therapies may be promising approaches for neurodegenerative disorders, including glaucoma (Nafissi and Foldvari, 2015). Neurotrophins, such as BDNF, ciliary neurotrophic factor (CNTF), and glial cell-line derived neurotrophic factor (GDNF), increase the survival of RGCs. However, the rapid clearance of neurotrophins limits their application. Genome editing therapy, which transports edited living cells that persistently express neurotrophins, overcomes this limit. Renexus^®^ is an encapsulated cell therapy-based NT-501intravitreal implant in which the NT-501 contains a genetically modified retinal pigment epithelium cell line that permanently secretes CNTF (Emerich and Thanos, 2008). NT-501 is being evaluated in two phase II clinical trials for the treatment of glaucoma (NCT02862938, NCT04577300). Osborne et al. designed a novel AAV gene therapy (AAV2 TrkB-2A-mBDNF) that not only increased BDNF level but also exerted long-term neuroprotection by increasing expression of the BDNF receptor (TrkB) within the inner retina (Osborne et al., 2018). In addition to neurotrophins, anti-axon retraction, anti-inflammation treatment, anti-apoptosis treatment, antioxidation treatment, and gene transfer of MAX, BRN3B, Hsp-70, PDEF, and EpoR76E also have the ability to protect RGC survival. Based on the finding that decreased content of the transcription factor Myc-associated protein X (MAX) is associated with degeneration of RGCs, a recent study demonstrated that overexpression of human MAX had a neuroprotective effect against RGC injury (Lani-Louzada et al., 2022).

Recently, mitochondrial dysfunction within the RGCs have been elucidated to be the one of the mechanisms of glaucoma (Abu-Amero et al., 2006). Reduced nicotinamide adenine dinucleotide (NAD+) levels have been closely related to mitochondrial dysfunction and have become features of neurodegenerative diseases including glaucoma. NAD + has been shown to be protective against axon degeneration *in vitro* and *in vivo* (Williams et al., 2017a). Various pathways are implicated to influence the NAD + levels, including NAD + synthesising enzyme and NAD + consuming enzymes. Upregulation of NAD + synthesising enzymes (QPRT, NADSYN1, NAPRT, NAMPT, NMRK, NMNAT) or downregulation of NAD + consuming enzymes such as SIRTS, PARPs, CD38/CD157, and SARM1 would result in increased NAD + levels. Subretinal injection of a normal copy of human NMNAT1 via AAV–mediated gene augmentation rescued retinal structure and function in Nmnat1-mutated mice (Greenwald et al., 2020). Overexpression of Nmnat1 in RGCs of D2 mice also prevented glaucomatous nerve damage in >70% of treated eyes (Williams et al., 2017b). On the other hand, suppression of SARM1 has been demonstrated to protect against mitochondrial dysfunction, leading to preservation of axon degeneration and retaining of visual function in an *in vivo* mouse model of RGC degeneration (Finnegan et al., 2022).

Except for gene therapy, direct supplement of NAD + have been evaluated, including treatment with the NAD + precursors nicotinamide (NAM), nicotinamide riboside (NR), or nicotinamide mononucleotide (NMN). Among them, oral administration of the NAM vitamin B3 in mice has very strong axonal protective effects (Williams et al., 2017b). Furthermore, NAD + has been applied in several clinical trials.

A recent small randomised trial of 57 glaucoma patients, demonstrated that oral NAM (1.five to three g/d) for 3 months significantly improved retinal function in glaucoma determined by photopic negative response (PhNR) parameters (Hui et al., 2020). Another clinical trial of 125 patients with POAG will be conducted to address whether daily nicotinamide riboside (NR) intake at 300 mg/day for 24 months has a neuroprotective effect in glaucoma patients (Leung et al., 2022).

### RGC and Optic Nerve Neuro-Regeneration

To overcome the irreversible loss of RGCs and optic nerve due to their inability of regeneration requires neuro-regenerative therapy. Gene therapies have been developed that modify axonogenesis-related genes, such as PTEN, SOCS3 (Sun et al., 2011), c-myc (Belin et al., 2015) and Nogo receptor (Dickendesher et al., 2012). There have also been a few attempts at using novel genome editing to promote axon regeneration. For instance, it has been demonstrated that the specific repression of PTEN by CRISPR/dCas9 promotes axon regeneration in rat neural crest-derived PC-12 cells (Moses et al., 2020).

As a result of the lack of regenerative ability of the human retina, the transplantation of living cells is the only way to recover vision loss after RGC death. The development of stem cell therapies allow for the replacement of degenerated and dead cells in glaucomatous retina using RGCs derived from stem cells, including human embryonic stem cells, iPSCs, and Müller glia cells. The discovery of Müller glia cell has generated great excitement in the field of cell replacement therapy as Müller glia cell can dedifferentiate to allow for their proliferation and differentiation into cell types that were damaged and thereby serve as retina progenitors.

Obstacles to neuro-regeneration that need to be overcome before successful application include RGCs being present at low proportions and hard-to-purify in stem cell-derived cultures and regenerated optic nerves have difficulty in exiting eyes and connecting with brain nuclei. Several proteins, non-coding RNAs (La Torre et al., 2013; Konar et al., 2020), and signals have been implicated in the difficulties associated with RGC transplantation. Therefore, cell replacement therapy may need the assistance of genome editing therapy and molecules that promote RGC survival and direct axon growth.

A recent study determined that down-regulation of a single RNA-binding protein, polypyrimidine tract-binding protein 1 (Ptbp1), by the *in vivo* delivery of the CRISPR system CasRx promotes expression of neuron-specific transcription factors, thereby increasing the efficiency of conversion of Müller glia cells to RGCs (Zhou et al., 2020). Notably, the Müller glia-derived RGCs established central projections to the brain and restored visual functions in an N-methyl-d-aspartate (NMDA)-induced retina injury mouse model. Another study revealed that trans-activation of the transcription factors Brn2, Ascl1, and Myt1l (BAM factors) by CRISPR/Cas9-based transcriptional activators can promotes epigenetic remodeling and gene over-expression, which thereby directly reprograms mouse embryonic fibroblasts to induced neuronal cells (Black et al., 2016). Furthermore, over-expression of Atoh7, an essential basic helix–loop–helix (bHLH) transcription factor for RGC differentiation, significantly increases the proportion of RGCs differentiated from Müller glia-derived stem cells (Song et al., 2013; Song et al., 2015). Similarly, Ngn2 is also a pro-neuralbHLH transcription factor expressed in retinal progenitor cells throughout retinal neurogenesis (Hufnagel et al., 2010) and transduction of Ascl1, Brn3b, and Ngn2 promotes the conversion of mouse fibroblasts to RGCs (Meng et al., 2013).

As discussed above, genome editing therapy can assist cell replacement therapy. Recent studies have suggested that applying the proper chemicals can convert fibroblasts to photoreceptors without the need of stem cells, thus opening a timesaving and clinically easy avenue for cell replacement therapy (Mahato et al., 2020). It was revealed that a combination of five small molecules, including Wnt/β-catenin pathway inhibitor IWR1, Repsox (VCR) combined with FSK (VCRF), and Sonic hedgehog, taurine, and retinoic acid (STR), is able to convert mouse embryonic fibroblasts into functional chemically induced photoreceptor-like cells (CiPCs). Subretinal transplantation of CiPCs into animals of a rod degeneration mouse model leads to long-term improvement in pupil reflex and partial restoration of visual function. The underlying mechanism indicates that mitochondria-translocated axis inhibition protein 2 (AXIN2) induces increased generation of reactive oxygen species, activation of NF-κB, and upregulation of Ascl1, which leads to the conversion of fibroblasts to photoreceptors. Therefore, combining pharmacologic reprogramming with genome editing is a prospective approach for converting fibroblasts to RGCs in the treatment of glaucoma.

Recently, investigators performed single-cell RNA sequencing (scRNA-seq) to construct the gene regulatory networks controlling Müller glia reprogramming (Hoang et al., 2020). They first conducted RNA-seq, scRNA-seq, and ATAC-seq of NMDA-induced or light-induced damaged mouse, zebrafish and chick retinas to profile the changes in gene expression and chromatin accessibility. Ten modules of differentially expressed genes and Müller glia-expressed transcription factors were then obtained to construct the regulatory networks for mice and zebrafish. A significant number of genes were identified that are highly related in the resting, reactivity, and reversion to resting statuses in mice and related to progression of the neurogenic status in zebrafish. For instance, hmga1a, smarca5, and yap1 in zebrafish and fatty acid-binding proteins (FABPs) in chicks are essential in reactive Müller glia for neurogenesis. Furthermore, nuclear factor I (NFI) in mice maintains Müller glia quiescence and reverts reactive Müller glia to the resting status. Deletion of NFI relieves the neurogenic barrier and promotes the development of multiple Müller glia-derived retinal neurons, including RGCs.

Research on the regeneration of RGCs and optic nerve is an essential component of curing glaucoma, but a long way remains before it can be truly applied to the clinical setting. Currently, scRNA-seq technology can be used to identify molecular changes after damage at the cellular level and then combined with the CRISPR/Cas9 gene editing system, which can achieve remarkable intervention effects by precisely targeting the relative key molecules. It is believed that with the continuing development of sequencing technology and gene editing technology, the application of RGCs and optic nerve regeneration therapy will become possible in the clinic setting and will facilitate the complete cure of glaucoma.

### Other Therapies

As noted above, ocular biomechanical properties influence glaucoma susceptibility and progression. Therefore, targeting the fibrous layer consisting of the cornea, sclera, and lamina cribrosa in patients with glaucoma is an alternative avenue for treatment. While genome editing therapy targeting the sclera and lamina cribrosa has not yet been performed, corneal gene modification using the CRISPR/Cas9 system has recently been successfully executed in several disease models, including for Meesmann’s epithelial corneal dystrophy (Courtney et al., 2016) and Fuchs’ endothelial corneal dystrophy (Rong et al., 2020), and has even entered clinical trials for viral keratitis (NCT04560790). The investigation of glaucoma-related cross-linking proteins, such as lysyl oxidase (LOX)/lysyl oxidase-like 1 (LOXL1), tissue trans-glutaminase (TG2), and advanced glycation end products, will allow corneal gene therapy to be further developed into a promising treatment of glaucoma.

Finally, anti-neovascular gene therapies may be useful for the prevention of neovascularization and uveitis and the treatment of secondary glaucoma after cataract surgery. A few such gene therapies have already been developed. For example, genome editing of vascular endothelial growth factor A (Vegfa) and hypoxia-inducible factor 1-alpha (Hif1a) by CRISPR/CjCas9 *in vivo* is able to abolish angiogenesis in an age-related macular degeneration model, supporting the possibility of applying this approach for treating secondary glaucoma after cataract surgery.

## Limitations and Prospects

As we described, different genome editing therapies targeting different ocular components in glaucoma currently exist, but many limitations remain resulting in their limited application. First, the most important limitation is the safety concern regarding off-target effects. Gene modification beyond the pathogenic site would result in the disruption of normal genes and off-target mutations, which may result in oncogenesis. Several studies have raised concerns regarding the potential for AAV vectors to cause pro-oncogenic events in treating hematological system diseases (Logan et al., 2017; Dalwadi et al., 2021). However, in all of the clinical studies of ocular gene therapy reported to date, ocular malignancies have not been found after injection of AAV or lentiviral vectors. Second, complications of genome editing therapies include gene therapy associated uveitis, there is a growing number of studies reporting immune responses and intraocular inflammation (Bainbridge et al., 2015; Tummala et al., 2021) and/or loss of efficacy after ocular delivery of clinical grade AAV (Bainbridge et al., 2015; Boyd et al., 2016). Besides, precise modification of the chosen gene may cause disappointing and detrimental outcomes due to the partial understanding of the complicated gene crosstalk. One example is that a clinical trial of 312 macular edema participants reported that 8.0% had IOP elevation more than 10 mmHg after intravitreal anti-VEGF injections (Aref et al., 2021), which reminds us to be cautious to apply anti-VEGF treatment for secondary glaucoma after cataract surgery as mentioned above. Third, many of the novel gene therapies discussed in this review were administrated simultaneously or even prior to the onset of optic nerve damage. In most cases of glaucoma, irreversible damage occurs before medical intervention is administered (Harvey et al., 2002). The efficacy of the gene therapies after damage occurs is unknown. Fourth, although AAV2 is considered the most efficient delivery system for gene therapy in rodents as mentioned above, transduction of RGCs is still largely inefficient by AAV2 in both large animals and humans (Ramachandran et al., 2017), which limits clinical applications of gene therapy for inner retinal diseases including glaucoma. Fifth, the specific disease mechanisms of glaucoma and pathways involved in its pathogenesis have not been fully elucidated. Therefore, the precise genome editing needs require further investigation. Finally yet importantly, the ethical, legal, and social implications of germline editing are always controversial topics and under continuous debate.

In summary, genome editing therapy has advanced greatly in recent years and has enormous potential in the treatment of glaucoma. With technological advancements and the obstacles addressed, genome editing will surely become a promising therapy for treating patients with glaucoma and will be widely applied in clinical practice.
